# Weight, Height, Breath

**Published:** 1871-06

**Authors:** 


### WEIGHT, HEIGHT, BREATH.

Fat is a disease; yet most persons want to weigh more. How much ought a man to weigh to be in perfect health? What is my healthful weight?

How much breath ought a man to have? how much of lungs to indicate that they are perfectly sound, working healthfully and well? Scientific men, and most notably Mr. Hutchinson of England, the inventor of the spirometer, or breath measurer, have made many thousands of measurements and weights, with the results that from five feet and upwards, the healthy weight of a man, giving the proper proportion of flesh, is five pounds of flesh for every additional inch in height.

The healthful amount of breath is so much for one five feet high, and for every additional inch in height eight additional cubic inches of air, or lung capacity for holding air, is required. That is, if a man is five feet high, he should weigh a hundred and fifteen pounds; if he is five feet and one inch, he should weigh a hundred and twenty pounds.

If a man is five feet high, he should be able to draw in or breathe out at a single inspiration or expiration one hundred and sixty-six cubic inches of air; if he is five feet one inch, then his lung capacity should be one hundred and seventy-four. In the light of these pretty well ascertained facts, the following table is prepared: —

| Height. | Weight. | Breath. |
|---|---|---|
| 5 feet. | 115 lbs. | 166 inches. |
| 5 " 1 inch. | 120 " | 174 " |
| 5 " 2 inches. | 125 " | 182 " |
| 5 " 3 " | 130 " | 190 " |

| HEIGHT. | WEIGHT. | BREATH. |
|---|---|---|
| 5 feet 4 inches. | 135 lbs. | 198 inches. |
| 5 " 5 " | 140 " | 206 " |
| 5 " 6 " | 145 " | 214 " |
| 5 " 7 " | 150 " | 222 " |
| 5 " 8 " | 155 " | 230 " |
| 5 " 9 " | 160 " | 238 " |
| 5 " 10 " | 165 " | 246 " |
| 5 " 11 " | 170 " | 252 " |
| 6 " | 175 " | 260 " |

These are the relative proportions of strong, robust, healthy men in England. Americans as a class have less flesh and more breath. As persons get old, the height declines, and also the breath, and still may be healthy; these are as yet approximative statements, and should be regarded as general or average proportions, which may be modified by more extended observations.

